# ﻿Revision of the genus *Alulacris* Zheng, 1981 (Orthoptera, Acrididae, Melanoplinae) with proposals of new synonyms

**DOI:** 10.3897/zookeys.1253.158995

**Published:** 2025-09-24

**Authors:** Yi Zhang, Benyong Mao, JoVonn G. Hill, Jianhua Huang

**Affiliations:** 1 Key Laboratory of Forest Bio-Resources and Integrated Pest Management for Higher Education in Hunan Province, Central South University of Forestry and Technology, Changsha 410004, China Central South University of Forestry and Technology Changsha China; 2 College of Agriculture and Biological Science, Dali University, Dali, Yunnan 671003, China Dali University Dali China; 3 Mississippi Entomological Museum, Department of Molecular Biology, Biochemistry, Entomology and Plant Pathology, Mississippi State University, Mississippi, USA Mississippi State University Mississippi United States of America

**Keywords:** Acrididae, Melanoplinae, new synonym, redescription, *
Tonkinacris
*

## Abstract

The genus *Alulacris* Zheng, 1981 is revised based on examination of type and non-type material of all known *Alulacris* species. As a result, two new junior synonyms are proposed: *A. yanshanensis* Mao et al., 2011, **syn. nov.** is synonymized with *A. shilinensis* (Zheng, 1977) and *A. nigristriatis*Zheng et al., 2013, **syn. nov.** is synonymized with *Tonkinacris sinensis* Chang, 1937. The morphology of *A. shilinensis* and *T. sinensis* are redescribed and illustrated. The reliability of quantitative traits for defining new species and the importance of the accurate generic assignment in determining new species are briefly discussed.

## ﻿Introduction

*Alulacris* Zheng, 1981 is endemic to China and belongs to the tribe Podismini Jacobson, 1905 (Orthoptera, Acrididae, Melanoplinae). It was established to contain a single species, *Pseudogerunda shilinensis* Zheng, 1977 (Zheng 1981). *Alulacris* is thought to be most closely related to the genus *Circocephalus* Willemse, 1928, but can be separated from the latter by male with tegmina extending to the second abdominal tergite and partially contiguous dorsally in most individuals. In female of *Alulacris* species, the tegmina sometimes reach or exceed the posterior margin of the second abdominal tergite but do not reach the middle of the third. Additional distinguishing characters for *Alulacris* species include the extremely vestigial hind wings and the small furculae on the tenth abdominal tergite.

To date, there are three known species of *Alulacris* (Zheng 1977, 1981, 1985, 1993; Yin et al. 1996; Li and Xia 2006; Mao et al. 2011a; Zheng et al. 2013; Cigliano et al. 2025). Mao et al. (2011a) described the second species, *A. yanshanensis*Mao et al., 2011a, based on a single holotype female. Zheng et al. (2013) subsequently described the third species, *A. nigristriatis*Zheng et al., 2013. Zheng (1981) did not assign *Alulacris* to a specific tribe or subfamily, but it has since been unambiguously considered as a member of the Podisminae sensu Li and Xia (2006). Xu et al. (2021) clarified the phylogenetic position of *Alulacris* based on complete mitogenome data, indicating that it is genetically most closely related to, and morphologically very similar to, the genus *Yunnanacris* Chang, 1940. However, the relationship among *Alulacris* species is still unclear.

*Tonkinacris* Carl, 1916 is a small group of grasshoppers with *T. decoratus* Carl, 1916 as type species and six known species from China, Vietnam and South Japan (Ryukyu Islands) (Carl 1916; Chang 1937; Li 1986; Li et al. 1991; Ito 1999; Cigliano et al. 2025). The distribution ranges of *Tonkinacris* species vary significantly. *Tonkinacris sinensis* Chang, 1937 has the broadest distribution, extending from Vietnam to Central China. *Tonkinacris decoratus* is distributed mainly in south Guangxi and north Vietnam with discrete records in central Guangxi, northwest Hunan and southwest Hubei. *Tonkinacris damingshanus*Li et al., 1991 and *T. meridionalis* Li, 1986 are both endemic to China and are known only from the type localities. Additionally, *Tonkinacris ruficerus* Ito, 1999 and *T. yaeyamaensis* Ito, 1999 are restricted to the Ryukyu Islands, Japan (Wang et al. 2021).

Although *Tonkinacris* species were the subject of a series of studies related to their taxonomy, phylogeny, and species delimitation (Wang et al. 2021), the phylogenetic relationship of *Tonkinacris* with its close relatives, the internal phylogeny within the genus, and the validity of *T. damingshanus* still require clarification.

Regarding the differential diagnoses of *Alulacris* species, both *A. yanshanensis* and *A. nigristriatis* were compared with *A. shilinensis* when described as new species (Mao et al. 2011a; Zheng et al. 2013). According to the original description, *Alulacris yanshanensis* can be distinguished from *A. shilinensis* by the following features. (1) The frontal ridge in female *A. yanshanensis* is shallowly sulcated only near antennae with weakly expanded lateral margins between antennae and obsolete near clypeus. In contrast, female of *A. shilinensis* has a sulcated frontal ridge throughout, with complete parallel lateral margins. (2) The interocular distance in female *A. yanshanensis* is slightly narrower (0.7 mm) and ~ 0.3× the longitudinal diameter of the eyes, whereas in female *A. shilinensis*, it is broader (0.9 mm) and ~ 0.4× the longitudinal diameter of the eyes. (3) In female *A. yanshanensis*, the posterior transverse sulcus of pronotum is located at the hind part of the pronotum, with the prozona being 1.5× as long as the metazona. In contrast, in female *A. shilinensis*, the sulcus is positioned near the middle of the pronotum, with the prozona ~1.22× as long as the metazona. (4) The tegmina of female *A. yanshanensis* are broader and 1.94× as long as maximum width, but in female *A. shilinensis*, they are narrower and 2.23× as long as maximum width. (5) The lower knee lobes of the hind femur are dark olivaceous in *A. yanshanensis* but black in *A. shilinensis* (Mao et al. 2011a).

*Alulacris nigristriatis* differs from *A. shilinensis* mainly as follows. (1) The male cercus is conical and distinctly constricted near apex in *A. nigristriatis*, whereas it is elongate with only a slight narrowing at apex in *A. shilinensis*. (2) The male tenth abdominal tergite bears mastoid furculae in *A. nigristriatis*, but small circular furculae in *A. shilinensis*. (3) In *A. nigristriatis*, the tegmina are yellowish brown with a black longitudinal stripe in the middle, in female just reaching the posterior margin of the second abdominal tergite and in male slightly surpassing it, but in *A. shilinensis* the tegmina are dark brown and unicolorous, and only extend to the middle of the second abdominal tergite in male. (4) *Alulacris nigristriatis* has a black post-ocular stripe, but in *A. shilinensis* the post-ocular stripe is lacking. (5) The hind tibiae are black-brown in *A. nigristriatis* but yellowish blue in *A. shilinensis* (Zheng et al. 2013).

When examining types of *Alulacris* species and some additional material of *A. shilinensis*, inconsistencies with the previous diagnoses were discovered. Therefore, we initiated a more thorough study of *Alulacris* and its close relative, *Tonkinacris*, to evaluate whether the current species hypotheses are supported. After a careful comparison of relative taxa, we found no significant difference between *A. shilinensis* and *A. yanshanensis* as well as between *A. nigristriatis* and *T. sinensis*. Consequently, we consider *A. yanshanensis* as a junior synonym of *A. shilinensis*, and consider *A. nigristriatis* as a junior synonym of *T. sinensis*. The morphology of *A. shilinensis* and *T. sinensis* are redescribed and illustrated.

## ﻿Materials and methods

We examined the types of all *Alulacris* species as well as additional material of *A. shilinensis* and *T. sinensis*. Photographs of the dried specimens and male genitalia were taken by the first author using a Nikon D600 digital camera or Leica DFC 5500 system, and the images were stacked using Helicon Focus v. 6.0 (https://www.heliconsoft.com/heliconsoft-products/helicon-focus/). The terminology for morphology follows Uvarov (1966) and Storozhenko et al. (2015). The terminology of male genitalia follows Dirsh (1956) and Woller and Song (2017).

The measurements generally used for grasshoppers are defined as below:

**BL** body length, the length from the apex of fastigium to the apex of subgenital plate;

**PNL** pronotum length, the length from the anterior margin of pronotum to the posterior margin;

**TL** tegmen length, the length from the base of tegmen to the apex;

**TW** tegmen width, the maximum width of tegmen;

**HFL** hind femur length, the maximum distance from the base of hind femur to the apex.

Specimens were examined from the Insect Collection of Central South University of Forestry & Technology in Changsha, China (**CSUFT**), with Jianhua Huang serving as curator; Dali University (**DU**) in Yunnan, China, with Benyong Mao as curator; the Museum of Zoology at Shaanxi Normal University (**SNU**) in Xi’an, China, with Liliang Lin as curator.

The quantitative data were measured under a stereomicroscope using an ocular micrometer. The two-sample Wilcoxon test (also known as “Mann-Whitney” test) were performed using the function “wilcox.test()” in the “stats” package of R 4.4.0.

## ﻿Results

### ﻿Reexamination of diagnostic characters between *Alulacris shilinensis* and *Alulacris yanshanensis*

Since three of five diagnostic characters used to delineate *A. yanshanensis* were quantitative characters (Mao et al. 2011a), we measured a total of 11 quantitative characters for both *A. shilinensis* and *A. yanshanensis* and calculated five related indices (Table 1). The result showed that most measurements of *A. yanshanensis* fell into the 95% confidence interval of those of *A. shilinensis*. Furthermore, all characters showed no significant difference between *A. shilinensis* and *A. yanshanensis* in the two-sample Wilcoxon test (Table 1).

**Table 1.** Zheng Statistical significance test of measurements of quantitative characters and some related ratios between *A. shilinensis* and *A. yanshanensis*.

Sample	BL	PNL	TL	TW	HFL	IOD	LDE	TDE
As_1	22.35	5.20	5.70	3.00	12.50	0.80	2.58	1.76
As_2	21.75	5.25	5.50	3.30	11.75	0.75	2.52	1.80
As_3	22.20	5.25	5.45	3.30	12.50	0.62	2.54	1.76
As_4	23.00	5.55	5.32	2.90	12.30	0.70	2.52	1.80
As_5	21.31	4.51	5.35	2.85	11.89	0.90	2.26	1.77
As_6	22.90	5.40	6.10	3.03	12.51	1.00	2.55	1.73
As_7	21.87	5.27	5.42	2.87	12.03	0.82	2.48	1.61
As_8	21.93	4.77	5.61	2.91	10.78	0.93	2.31	1.71
As_9	21.96	5.18	4.92	1.94	12.18	0.91	2.52	1.88
As_10	22.10	5.39	5.49	2.71	11.37	0.89	2.28	1.82
As_11	21.90	4.74	5.26	2.49	11.21	0.95	2.19	1.59
As_12	22.15	5.45	6.68	2.88	12.53	0.90	2.45	1.82
As_mean	22.12 ± 0.92	5.16 ± 0.63	5.57 ± 0.88	2.85 ± 0.71	11.96 ± 1.14	0.85 ± 0.22	2.43 ± 0.26	1.75 ± 0.17
Ay_hf	22.64	5.05	5.72	2.70	12.05	0.91	2.07	1.53
W value	2	9	2	10	6	3.5	12	12
p value	0.4615	0.5034	0.4615	0.3489	1.0000	0.592	0.1394	0.1399
Sample	SOFL	PZL	MZL	IOD/LDE	LDE/TDE	LDE/SOFL	TL/TW	PZL/MZL
As_1	1.70	3.05	2.15	0.31	1.47	1.52	1.90	1.42
As_2	1.64	3.00	2.25	0.30	1.40	1.54	1.67	1.33
As_3	1.55	3.10	2.15	0.24	1.44	1.64	1.65	1.44
As_4	1.60	3.20	2.35	0.28	1.40	1.57	1.83	1.36
As_5	1.50	2.56	1.95	0.40	1.28	1.51	1.88	1.31
As_6	1.44	3.12	2.28	0.39	1.47	1.77	2.01	1.37
As_7	1.37	3.03	2.24	0.33	1.54	1.81	1.89	1.35
As_8	1.42	2.84	1.93	0.40	1.35	1.63	1.93	1.47
As_9	1.34	3.04	2.14	0.36	1.34	1.88	2.54	1.42
As_10	1.55	3.01	2.38	0.39	1.25	1.47	2.03	1.26
As_11	1.37	2.67	2.07	0.43	1.38	1.60	2.11	1.29
As_12	1.49	2.93	2.52	0.37	1.35	1.64	2.32	1.16
As_mean	1.50 ± 0.22	2.96 ± 0.37	2.20 ± 0.34	0.35 ± 0.11	1.39 ± 0.16	1.63 ± 0.25	1.98 ± 0.50	1.35 ± 0.17
Ay_hf	1.54	2.96	2.09	0.44	1.35	1.34	2.12	1.42
W value	5	8	9	0	8	12	2	3
p value	0.8934	0.7692	0.5034	0.14	0.69	0.14	0.46	0.50

* Note: The sample codes from“As_1” to “As_12” represent the measured female individuals of *A. shilinensis*, and “Ay_hf” refers as to the holotype female of *A. yanshanensis*. The acronyms for measurements are as follows: BL–Body length, PNL–Pronotum length, TL–Tegmina length, TW–Tegmen width, HFL–Hind femur length, IOD–Interocular distance, LDE–Longitudinal diameter of eyes, TDE–Transversal diameter of eyes, SOFL–Length of subocular furrow,PZL–Prozona length, MZL–Metazona length, IOD/LDE–Ratio of IOD to LDE, LDE/TDE–Ratio of LDE to TDE, LDE/SOFL–Ratio of LDE to SOFL, TL/TW–Ratio of TL to TW, PZL/MZL–Ratio of PZL to MZL.

**Figure 1.**
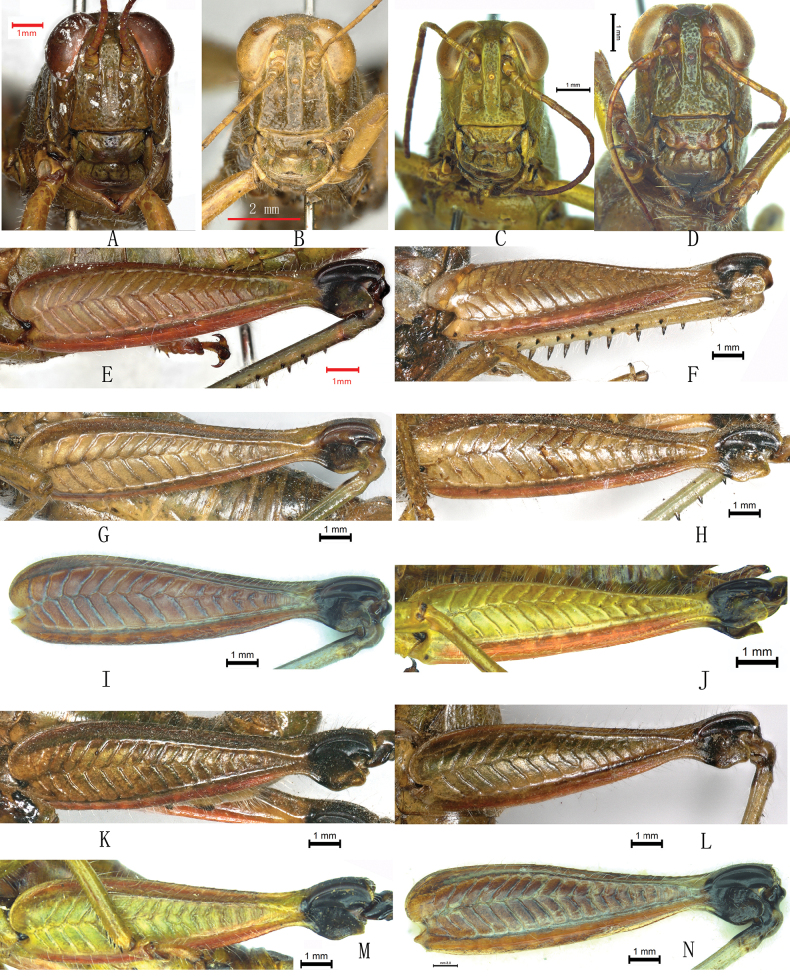
Head and hind femur of *Alulacris* species. A–D. Head in frontal view; A. Holotype female of *A. yanshanensis*; B. Holotype female of *A. shilinensis*; C, D. Non-type females of *A. shilinensis*; E–N. Hind femur in lateral view; E. Holotype female of *A. yanshanensis*; F. Holotype female of *A. shilinensis*; G–J. Non-type females of *A. shilinensis*; K–N. Males of *A. shilinensis*.

According to the original description (Mao et al. 2011a), besides the three quantitative characters which can distinguish *A. yanshanensis* from *A. shilinensis*, the frontal ridge and the color of lower knee lobes of hind femur were noted to be useful in the identification of *A. shilinensis*. However, after a careful comparison of holotype female of *A. yanshanensis* to type and non-type material of *A. shilinensis*, it was found that there was no detectable difference in these two qualitative characters between the two species. In both species, the lateral margins of frontal ridge are weakly expanded between antennae with minor variation among individuals (Fig. 1A–D). While the lateral margins of frontal ridge vanish near clypeus in *A. yanshanensis* (Fig. 1A), they are distinct throughout in holotype female of *A. shilinensis* (Fig. 1B) and vanish near the clypeus in some individuals (Fig. 1C, D). The length of sulcus of frontal ridge varies in *A. shilinensis*, i.e., sulcated thoroughly (Fig. 1B) or nearly thoroughly (Fig. 1C) in some individuals but only partially in other individuals (Fig. 1D) just as in *A. yanshanensis* (Fig. 1A). As for the color of lower knee lobes of hind femur, it is really dark olivaceous and variegated with dark brown in *A. yanshanensis* (Fig. 1E), but varies in *A. shilinensis* from yellowish brown (Fig. 1F) through olivaceous (Fig. 1G–J) to dark brown (Fig. 1K, L) or even pure black (Fig. 1M, N).

**Figure 2.**
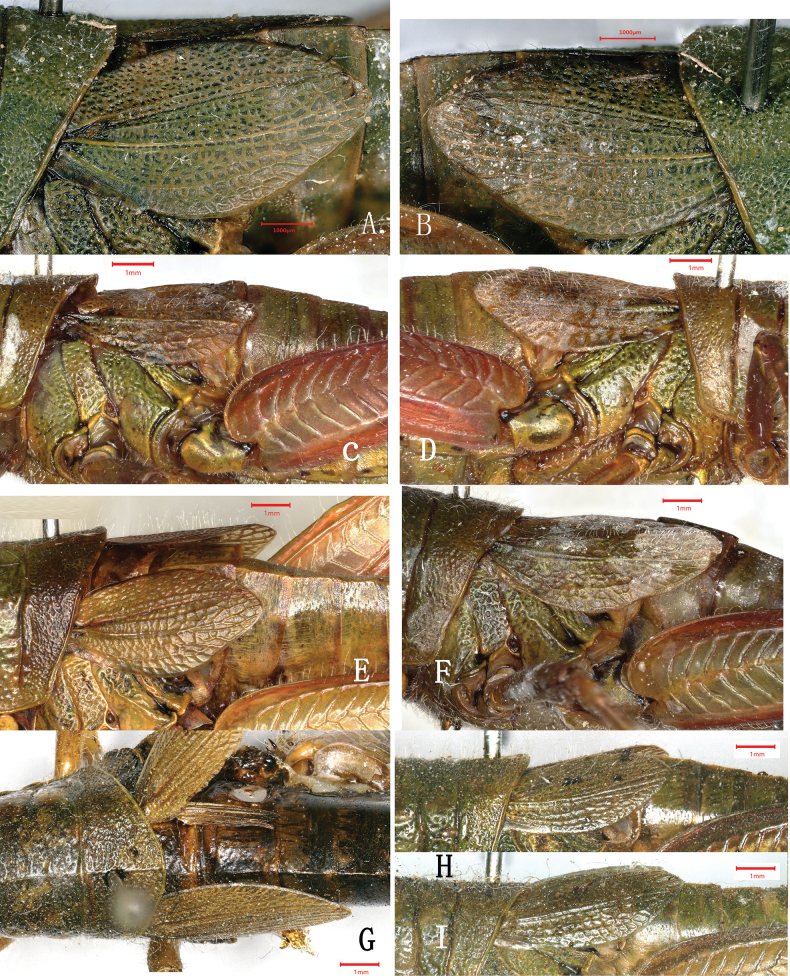
Tegmina of *Alulacris* spp. A, B. Holotype female of *A. yanshanensis*; C–F. Non-type female of *A. shilinensis*; G. Holotype female of *A. shilinensis*; H, I. Male of *A. shilinensis*.

Variation is also observed in the length of tegmina in both species. Although there is only the single holotype female available for *A. yanshanensis* to examine, it still displays a minor variation in length between left and right tegmina, with the left one slightly exceeding but the right one slightly not reaching the posterior margin of the second abdominal tergite (Fig. 2A, B). The range of variation in length of tegmen is much broader in females of *A. shilinensis*, with the shortest one just slightly exceeding the posterior margin of the first abdominal tergite (Fig. 2C), the moderate ones reaching the middle (Fig. 2E) or nearly reaching the posterior margin (Fig. 2F) of the second abdominal tergite, and the longest one exceeding the posterior margin of the second abdominal tergite (Fig. 2G). Similar variation pattern was also observed in male of *A. shilinensis* (Fig. 2H, I). The variation in tegmen length is also observed between the left and right tegmina of the same individual in *A. shilinensis*. In one female, the left tegmen just slightly exceeds the posterior margin of the first abdominal tergite (Fig. 2C) but the right one reaches the two thirds of the second abdominal tergite (Fig. 2D).

### ﻿Reexamination of diagnostic characters for generic assignment of *Alulacris nigristriatis*

**Figure 3.**
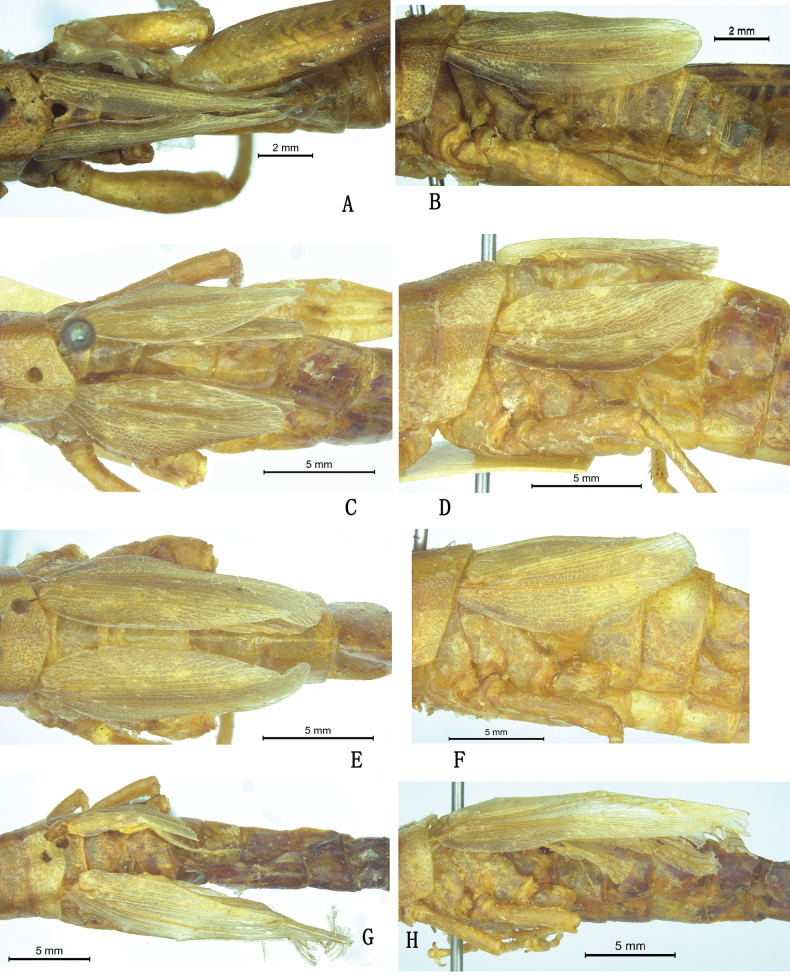
Tegmina of *Alulacris nigristriatis*. A, B. Holotype male; C–H. Paratype females showing the variation in tegmen length.

According to the modern classification of the subfamily Melanoplinae (Otte 1995; Cigliano et al. 2025), the genus *Alulacris* is a member of the subtribe Podismina Jacobson, 1905 in the tribe Podismini Jacobson, 1905, while *Tonkinacris* is a type genus of the subtribe Tonkinaridina Ito, 2015 in the tribe Podismini Jacobson, 1905. Chinese acridologists usually divide the Chinese Melanoplinae into two groups based on the appearance of the tegmina (Li and Xia 2006). The group containing *Alulacris* has the tegmina dorsally separated or at most slightly touching each other, and extending only to the base of hind femur, and the other group involving *Tonkinacris* has the tegmina completely contiguous and reaching at least the middle of hind femur. Therefore, we hypothesize that the main morphological evidence for assigning the types representing *A. nigristriatis* to the group involving *Alulacris* is the reduced and broadly squamiform tegmina which exhibit a slight dorsal contiguity, exceed in males and just reach the posterior margin of the second abdominal tergite in females (Zheng et al. 2013). To clarify this placement, we reexamined the tegmina of the type series of *A. nigristriatis*. We found that the dorsal separation of the tegmina of these specimens might be an artifact caused by the deformation resulting from strong pressure during preservation in small plastic tubes because all of the four types were dramatically deformed. Regarding the tegminal length, there was significant variation among the specimens and the original description lacked precision. In the holotype male, the tegmina slightly surpass the posterior margin of the third abdominal tergite but not of the second one contrasting with the original reference (Zheng et al. 2013) (Fig. 3A, B). In two female paratypes, the tegmina just reach or do not reach the posterior margin of the second abdominal tergite (Fig. 3C–F), but in another female paratype, they are much longer and reach the middle of the fifth abdominal tergite (Fig. 3G, H).

Assigning the types of *A. nigristriatis* to the group involving *Alulacris* based on a variable or deformed character status seems unreasonable, especially as the possibly completely contiguous and longer tegmina demonstrate that *A. nigristriatis* should belong to the group with longer and dorsally contiguous tegmina. So we compared it with all genera of this group from China and found that it conforms with the diagnosis of *Tonkinacris* and showed no difference from *T. sinensis*.

### ﻿Revision of *Alulacris* Zheng, 1981

*Alulacris* Zheng, 1981: 65; Zheng 1985: 156; Zheng 1993: 113; Otte 1995: 268; Yin et al. 1996: 48; Li and Xia 2006: 258; Zhen et al. 2010: 251; Mao et al. 2011a: 93, 94; Xu et al. 2021: 2, 12.

**Type species.***Pseudogerunda shilinensis* Zheng, 1977.

**Generic diagnosis.** Body small to medium-sized and slightly robust. Head shorter than pronotum. Frons slightly oblique in lateral view, with frontal ridge straight and distinctly sulcate. Eyes oval. Antennae filiform, slender and elongate, exceeding posterior margin of pronotum. Pronotum cylindrical with rugose, puncture and long hair; anterior margin straight and posterior margin broadly convex; median carina distinct only on metazona; lateral keel absent. Prosternal spine conical with a rounded apex. Lateral lobes of mesosternum broadly separate, slightly broader than long, lateral lobes of metasternum nearly contiguous in male and distinctly separated in female. Tegmina distinctly reduced and broadly squamiform with long hair, narrowly separated dorsally in most individuals or partially contiguous dorsally in a few individuals, just reaching the base of hind femur (or the middle of the second abdominal tergite in male and exceeding the posterior margin of the second abdominal tergite but not reaching the middle of the third abdominal tergite in female). Hind wing extremely vestigial. Hind femur with upper basal lobe of outer surface distinctly longer than lower basal lobe, upper median keel smooth and sharply spinous apically, lower genicular lobe rounded apically. Hind tibia without outer apical spine. Tarsus with large arolium exceeding apex of claw. Tympanum large and rounded. Dorsum of abdomen with distinct median keel. Tenth abdominal tergite with a pair of small circular furculae. Supra-anal plate triangular. Cerci in male laterally compressed, lamellate with apex broadly rounded, only slightly narrower than base but not strongly constricted. Subgenital plate of male short conical with pointed apex. General outline of epiphallus trapezoidal in dorsal view; ancora long and lamellate, as high as the anterior projections, strongly curved ventrally, with bluntly rounded apex; lophi vertical, large, lamellate and broadly auriform, with broadly rounded apical margins; phallic complex with apical valves of penis and valves of cingulum slender and lamellate. Outer margin of upper valves of ovipositor with small blunt denticles.

**Distribution.** China (Yunnan).

**Species composition.***Aululacris shilinensis* (Zheng, 1977) (monotypic genus).

#### ﻿ Alulacris shilinensis

Taxon classificationAnimaliaOrthopteraAcrididae

(Zheng, 1977)

C2744249-B085-5884-9C7A-B4D835CA791C

Figs 1, 2, 4, 5A–D

Pseudogerunda shilinensis Zheng, 1977: 305, 311. Type locality: China (Yunnan: Shilin).
Alulacris shilinensis (Zheng); Zheng 1981: 60, 65; Zheng 1985:157; Zheng 1993: 114; Otte 1995: 268; Li and Xia 2006: 260; Mao et al. 2011a: 94, 302; Zheng et al. 2013: 90, 91; Xu et al. 2021: 4, 13.
Alulacris yanshanensis Mao et al., 2011a: 95, 302. Type locality: China (Yunnan: Yanshan). Syn. nov.

##### Type material examined.

***A. shilinensis*.** China • ***Holotype*** female; Yunnan, Kunming, Shilin, Lunan; July 1974; Sumin Zheng leg. (SNU). • 1 ♂ 1 ♀; Yunnan, Kunming, Shilin; 21 August 1978; Zhemin Zheng leg. (SNU). • 2 ♂ 1 ♀; Yunnan, Kunming, Shilin; 12 September 1980; Zhenmin Lian leg. (SNU). • 6 ♂ 4 ♀; Yunnan, Kunming, Shilin County, Shilin Landscape area; 24°48'19"N, 103°19'16"E; 1778 m; 15 August 2017; Bing Jiang Leg. (CSUFT). • 1 ♂ 1 ♀; Yunnan, Kunming, Shilin; 1 August 1985; Yuan Huang leg. (SNU). • 5 ♂ 6 ♀; Yunnan, Kunming, Shilin; 1700 m; 15 August 2017; Benyong Mao leg. (DU). • 3 ♂ 2 ♀; Yunnan, Xinping County, Xinhua; 23 August 2019; Benyong Mao leg. (DU).

***A. yanshanensis*.** China • ***Holotype*** female; Yunnan Province, Wenshan Zhuang and Miao Autonomous Prefecture, Yanshan County; 9 November 2003; Jishan Xu leg. (DU).

##### Morphology.

**Figure 4.**
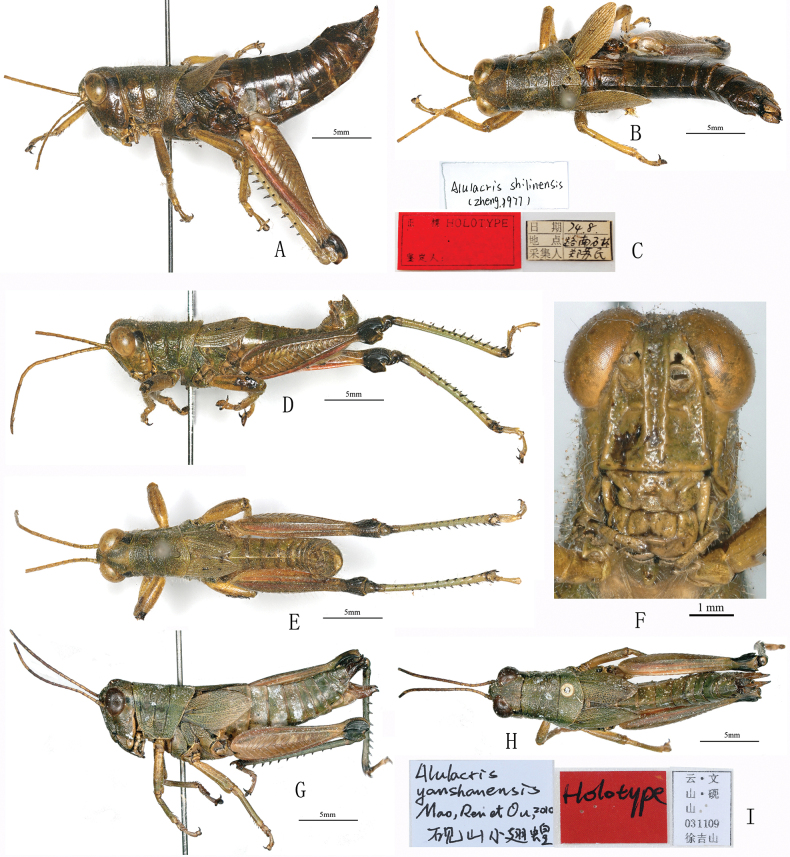
Habitus of *Alulacris* spp. A–F. *A. shilinensis*; A, B. Holotype female in lateral and dorsal views; C. Labels of holotype female; D, E. male in lateral and dorsal views; F. Head in frontal view; G–I. *A. yanshanensis*; G, H. Holotype female in lateral and dorsal views; I. Labels of holotype female.

**Figure 5.**
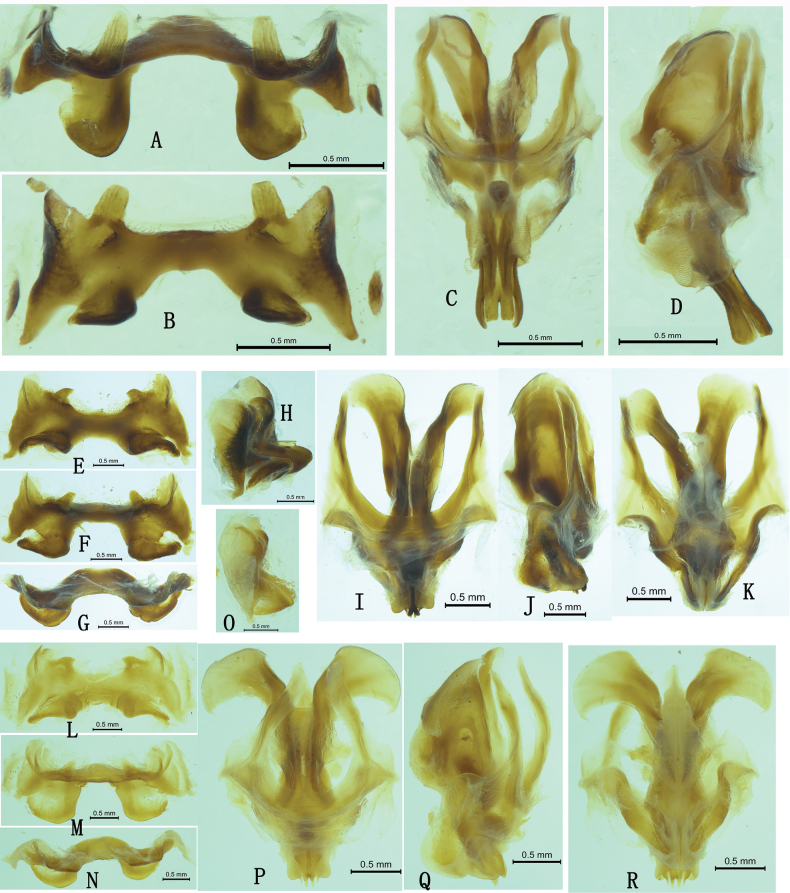
Male genitalia of *Alulacris shilinensis*, *Tonkinacris sinensis* and *Alulacris nigristriatis*. A–D. *Alulacris shilinensis*; A, B. Epiphallus in dorsofrontal and dorsal views; C, D. Phallic complex in dorsal and lateral views; E–K. *Tonkinacris sinensis*; E–H. Epiphallus in dorsal, dorsofrontal, frontal and lateral views; I–K. Phallic complex in dorsal, lateral and ventral views; L–R. *Alulacris nigristriatis*; L–O. Epiphallus in dorsal, dorsofrontal, frontal and lateral views; P–R. Phallic complex in dorsal, lateral and ventral views.

**Male.** Body small to medium-sized, covered with s sparse hairs. Head shorter than pronotum. Vertex short and strongly oblique, fastigium broadly connected with frontal ridge. Frons slightly inclined backward in profile view. Frontal ridge straight, with distinct longitudinal sulcus throughout and nearly parallel lateral margins which slightly expand near the antennae in some individuals (Fig. 4F). Antennae filiform, extending beyond posterior margin of pronotum. Eyes oval, with a longitudinal diameter 1.3–1.5× as long as transversal diameter and 1.5–2.2× as long as the subocular furrow. Interocular distance ~ 0.4 mm. Pronotum cylindrical, rugulose, and punctate; anterior margin relatively straight or slightly concave in the middle; posterior margin roundly convex; median carina distinct in metazona; lateral carina absent; posterior transversal sulcus located near the middle of pronotum; prozona 1.4–1.7× as long as the metazona; lower margin of the lateral lobe roundly and bluntly convex near the middle, with broadly rounded anterior angle and acutely rounded posterior angle. Prosternal process conical, with a rounded apex. Lateral lobes of mesosternum slightly broader than long, with rounded inner margins. Lateral lobes of metasternum nearly contiguous with each other posteriorly. Tegmina broadly squamiform with sparse long hair, reaching the middle (Fig. 2H) or slightly exceeding the posterior margin (Fig. 2I) of the second abdominal tergite, partially contiguous dorsally in most individuals (Fig. 4E) or slightly separate in a few individuals. Hind wings extremely vestigial. Hind femora with upper basal lobe of outer surface distinctly longer than lower basal lobe, upper median keel smooth and sharply spinous apically, lower genicular lobe rounded apically. Hind tibia with 10 or 11 spines at both inner and outer margins; outer apical spine absent. Tarsus with large arolium exceeding apex of claws. Tympanum large and rounded. Dorsum of abdomen with distinct median keel. The tenth abdominal tergite with a pair of small circular furculae. Supra-anal plate broadly triangular and longitudinally sulcate in the middle, with two tubercles at basal lateral margins. Cerci broadly laterally compressed and lamellate, not reaching apex of supra-anal plate, with broad base and slightly rounded apex. Subgenital plate short conical with pointed apex and long hair. Epiphallus with a broad undivided bridge; general outline trapezoidal in dorsal view; bridge a little broad; lateral margins oblique inwards; ancora long and lamellate, as high as the anterior projections (Fig. 5B), strongly curved ventrally, with bluntly rounded apex; anterior projection triangular and posterior projection long conical; lophi vertical, large, lamellate and broadly auriform, with broadly rounded apical margins. Phallic complex with apical valves of penis and valves of cingulum slender and lamellate. Valves of cingulum deeply emarginate in the middle of apex. Apodemes elongate, nearly reaching apex of the basal valves of penis.

**Female.** Similar to male, except for the following. Lateral margins of frontal ridge complete (Fig. 1B) or absent near the clypeus (Fig. 1A, C, D). Interocular distance relatively wider, ranging from 0.6 to 1.0 mm. Prozona 1.2–1.4× as long as metazona. Lateral lobes of metasternum distinctly separated. Tegmina at least reaching the posterior margin of the first abdominal tergite (Fig. 2C), or exceeding the posterior margin of the second abdominal tergite (Fig. 2G), but not reaching the middle of the third abdominal tergite, slightly separated from each other dorsally in most individuals (Fig. 2B) but partially contiguous with each other dorsally in a few individuals (Fig. 2H). Epiproct elongated triangular, with a transverse sulcus in the middle and a longitudinal sulcus at basal half. Cerci shortly conical, only reaching half of epiproct. Outer margin of dorsal valves of ovipositor with small blunt denticles.

##### Coloration.

Body dark olivaceous. Hind femur with outer and upper sides green, and inner and lower sides orange-yellow to orange-red; lower knee lobe dark green, yellowish brown or black. Hind tibia bluish-green. Hind tarsus pale brown.

##### Measurements.

Male: BL: 16.0–17.0 mm; PNL: 4.0–4.5 mm; TL: 4.0–4.5 mm; HFL: 9.0–10.0 mm. Female: BL: 23.0–24.0 mm; PNL: 5.0–6.0 mm; TL: 5.5–6.0 mm; HFL: 13.0–14.0 mm.

##### Distribution.

China (Yunnan: Shilin, Yanshan, Xingping).

##### Biology.

No data on the biology of the species (Mao et al. 2011a).

##### Remark.

*Alulacris yanshanensis* was described only based on a single holotype female and differs from *A. shilinensis* mainly in five external morphological traits as mentioned in the Introduction, of which three are quantitative and two are qualitative. However, after a careful comparison of the type of *A. yanshanensis* with the type series of *A. shilinensis* as well as some additional material, no significant difference in both qualitative and quantitative characters were found between these two species (Table 1, Figs 1, 2). Therefore, *A. yanshanensis* is considered herein as a junior synonym of *A. shilinensis*.

### ﻿Revision of *Tonkinacris* Carl, 1916

*Tonkinacris* Carl, 1916: 485; Chang 1940: 38, 65; Bey-Bienko and Mistshenko 1951: 237; Mistshenko 1952: 397; Willemse 1957: 476; Xia 1958: 52; Zheng 1985: 172; Zheng 1993: 111; Otte 1995: 442; Yin et al. 1996: 708; Jiang and Zheng 1998: 122; Ito 1999: 505; Li and Xia 2006: 247; Ito 2015: 81; Wang et al. 2021: 1–17.

**Type species.***Tonkinacris decoratus* Carl, 1916.

**Figure 6.**
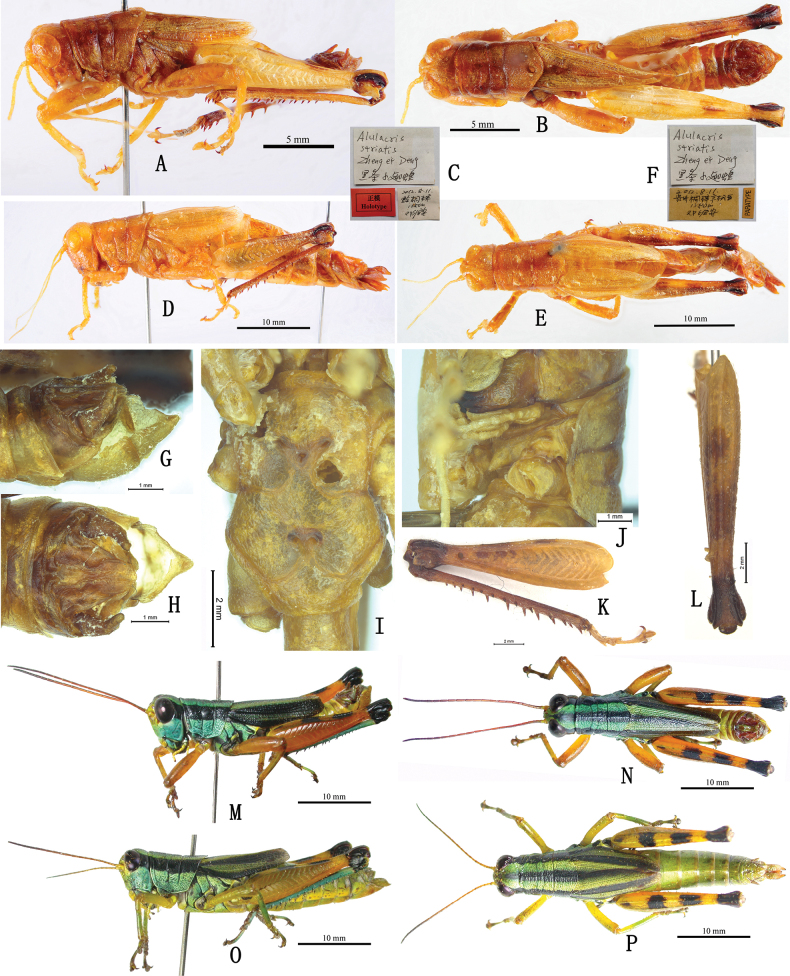
Habitus of *Alulacris nigristriatis* and *Tonkinacris sinensis*; A–L. Types of *A. nigristriatis*; A, B. Holotype male in lateral and dorsal views; C. Labels of holotype male; D, E. Paratype female in lateral and dorsal views; F. Labels of paratype female; M–P. Habitus of *T. sinensis*; M, N. Male in lateral and dorsal views; O, P. Female in lateral and dorsal views.

**Generic diagnosis.** Body medium-sized, well-proportioned and sparsely pubescent. Head large and shorter than pronotum; foveola absent. Eyes large and oval. Antennae slender and filiform, distantly exceeding posterior margin of pronotum. Pronotum cylindrical, slightly longer than broad; anterior margin slightly straight, posterior margin broadly rounded; median keel slightly low, lateral keel absent; all of three transverse sulci distinct and interrupting median keel. Prosternal process short conical and pointed apically, slightly oblique posteriorly. Mesosternum with lateral lobes broadly separated from each other and interspace between lateral lobes broad. Tegmina with longitudinal black stripes in the middle region, anterior and posterior margins, and without a series of dense and parallel short transverse veins in radial area; both tegmina and hind wings slightly reduced, usually reaching or exceeding the middle of hind femur (or at least exceeding the posterior margin of the third abdominal tergite in female and the posterior margin of the fourth to sixth abdominal tergite in male), completely contiguous dorsally. Hind femora without tooth in upper median keels; upper basal lobe of outer surface distinctly longer than lower basal lobe; upper and lower genicular lobes broadly rounded apically. Hind tibiae with nine or ten spines in outer margins, ectoapical spine absent. Abdomen with developed tympanum in each lateral side of the first segment; the tenth abdominal tergite in male with two small but distinct papillary furculae in the middle of posterior margin. Supera-anal plate long and triangular, longitudinally sulcate in the middle; apex slightly rounded. In male, cerci elongate, slightly curved apically to the inner and constricted apically (Fig. 6G, H); subgenital plate short and robust, with apex narrowly lamellate and truncate or pointed (Fig. 6H). In female, cerci straight and conical, subgenital plate rectangular, posterior margin triangularly protruded in the middle; upper valves of ovipositor long and lower valves with blunt denticles on the base of outer margins. General outline of epiphallus trapezoidal in dorsal view; ancora broadly lamellate, triangular, lower than anterior projections, with rounded apex strongly curved ventrally; lophi broad, lamellate and auriform, obliquely located along the inner margins of lateral plate; phallic complex with apical valves of penis and valves of cingulum relatively small and short.

**Distribution**. China, Japan.

**Species composition.** There are six known species in the genus *Tonkinacris*: *T. damingshanus*Li et al., 1991; *T. decoratus* Carl, 1916; *T. meridionalis* Li, 1986; *T. ruficerus* Ito, 1999; *T. sinensis* Chang, 1937; *T. yaeyamaensis* Ito, 1999.

#### ﻿ Tonkinacris sinensis

Taxon classificationAnimaliaOrthopteraAcrididae

Chang, 1937

934EED00-897B-52CD-A001-FA567BAD62CE

Figs 3, 5E–R, 6

Tonkinacris sinensis Chang, 1937: 191; Chang 1940: 67; Bey-Bienko and Mistshenko 1951: 252; Mistshenko 1952: 441; Xia 1958: 52; Zheng 1985: 173; Zheng 1993: 111; Jiang and Zheng 1998: 123; Li and Xia 2006: 248; Huang et al. 2013b: 1–17; Kim and Pham 2014: 56; Zhang et al. 2017: 147; Wang et al. 2021: 1–17. Type locality: China (Sichuan, Mt Omei).
Tonkinacris omei Rehn, 1938: 63; Mistshenko 1952: 399. Synonymized by Bey-Bienko and Mistshenko 1951: 253. Type locality: China (Sichuan, Mt Omei).
Alulacris nigristriatis Zheng et al., 2013: 90. Type locality: China (Guizhou, Tongzi). Syn. nov.

##### Type material examined.

***Alulacris nigristriatis.*** China • ***Holotype*** male, Guizhou Province, Tongzi, JingfengYa; 28°3'N, 106°8'E, 1250 m; 11 August 2012, Wei’an Deng leg. (SNU) • Paratypes, 3 ♀,data same as holotype (SNU).

##### Additional material examined.

***Tonkinacris sinensis***. China • 11 ♂ 13 ♀; Guangxi, Jingxi County, Diding; 23°6'56.5"N, 105°58'28.7"E; 800–900 m; broad-leaved forest; 8 August 2010; Jianhua Huang leg. (CSUFT) • 20 ♂ 23 ♀; Guangxi, Nanning City, Wuming District, Damingshan Nature Reserv; 4 September 2024; Jianhua Huang leg. (CSUFT) • 15 ♂ 15 ♀; Guangxi, Longzhou County, Wude Town, Sanlian Village; 15 July 2013; Tao Wei leg. (CSUFT) • 5 ♂ 7 ♀; Guangxi, Maoershan Natural Reserve (Xing’an County, Huajiang Town, Gaozhai Village); 21 August 2020; Zhilin Chen leg. (CSUFT) • 3 ♂ 5 ♀; Hunan Province, Yingzuijie Natural Reserve (Huitong County, Tuanhe Town, Xiangyang Village, Guixi); 2 August 2022; Jianhua Huang leg. (CSUFT) • Sichuan Province, Emeishan, Hongchunping; 12 August 2011, Ruigang Yang leg. (CSUFT) • 1 ♂ 1 ♀; Chongqing City, Nanchuan District, Jinfoshan; 21 August 2003; Caixia Yuan leg. (CSUFT).

##### Morphology.

**Male.** Body medium-sized and robust, with a few sparse pubescence. Head shorter than pronotum, nearly as long as or slightly longer than metazona of pronotum. Vertex short, inclined forwards, slightly triangular and longitudinally sulcate between eyes. Frons slightly oblique backwards in profile view, roundly connected with fastigium. Frontal ridge with incomplete longitudinal sulcus and nearly parallel lateral margins. Lateral facial keels distinct and straight. Antennae filiform, slender, reaching the base of hind femur, with median segments 3.5–5.5× as long as broad. Eyes oval, with longitudinal diameter 1.3–1.8× horizontal diameter, and 1.5–2.0× as long as subocular furrow. Interocular distance extremely narrow, shorter than the distance from anterior margin of eyes to the top of vertex. Pronotum cylindrical and densely punctate dorsally; anterior margin straight, slightly concave in the middle; posterior margin broadly rounded, distinctly protruding in an obtuse angle in the middle; median keel fine, distinct at metazona only; lateral keel absent; three transversal sulci all distinct, cutting off median keel; posterior transversal sulcus located behind the middle; prozona 1.2–1.4× as long as metazona. Prosternal process conical, slightly oblique backwards, with pointed or rounded apex. Mesosternal lobes broader than long, broadly separated, with inner margin broadly convex and interspace between mesosternal lobes goblet-shaped, longer than minimum width; metasternal lobes distinctly separated posteriorly. Tegmina reduced with narrow and angulate apex, contiguous dorsally, at least exceeding posterior margin of the third abdominal segment, but not reaching posterior margin of the fifth abdominal segment. Hind wings slightly shorter than tegmina. Fore and mid femora slightly enlarged. Hind femur proportioned, with upper median keel smooth and edentate. Hind tibia without outer apical spine, with 9–11 spines at inner and outer margins. Tarsus with large arolium, exceeding apex of claws. Tympanal organ large and round. Posterior margin of The tenth abdominal tergite with a pair of small papillary furculae. Cercus conical, broad basally, slightly narrowed in the middle, distinctly curved upwards and constricted at apical third, forming a broadly rounded angle at lower margin; apex bluntly rounded, not reach the top of supra-anal plate. Supra-anal plate long triangular, with a longitudinal sulcus in the middle which is extremely broad in the basal third and become very small in the middle third after a distinct constriction; apical third roundly depressed and thickly carinated around the basal two thirds of the depression; lateral area with a broad and longitudinal depression beside the median longitudinal sulcus and a narrower and shorter longitudinal sulcus along the lateral margins. Subgenital plate short conical with blunt and triangularly protruding apex; lateral margins nearly parallel at basal and then strongly constricted apically.

Epiphallus with an undivided bridge; general outline trapezoidal in dorsal view, lateral margins shallowly concave in the middle; anterior projections broad with rounded apical margins; posterior projections conical having a bluntly rounded apex; bridge relatively broad; ancora broadly lamellate, triangular, lower than anterior projections (Fig. 5E, L) with rounded apex strongly curved ventrally; lophi broad, lamellate and auriform, obliquely located along the inner margins of lateral plate. Phallic complex with apical valves of penis and valves of cingulum relatively small; rami of cingulum very developed, connected ventrally, forming a sheath enveloping the apical part of the penis and forming the whole apex of the aedeagus; apodemes horn-shaped, not reach the apex of basal valves of penis; basal valves of penis very broad.

**Female.** Similar to male, except for the following. Body larger than male. Vertex broadly rounded; interocular distance as long as the distance from anterior margin of eyes to apex of fastigium; eyes with longitudinal diameter 1.4–1.8× as long as horizontal diameter, and 1.4–1.7× as long as subocular furrow. Antennae slightly shorter than in male, reaching posterior margin of pronotum. Pronotum with median keel distinct throughout. Tegmen developed, exceeding the middle of the second abdominal tergite, but not reaching the anterior margin of the sixth abdominal tergite. Hind wing slightly shorter than tegmen. Hind femur well proportioned, with finely denticulate upper median keel. Hind tibia with 9–12 spines at outer margin. Supra-anal plate long and triangular, with a transversal sulcus near the middle and a complete median longitudinal groove, which is broad and deep at base and become narrower apically. Cercus conical, not curved. Dorsal and ventral valves of ovipositor relatively short and thick, crooked; outer margin of dorsal valves with indistinct denticles. Subgenital plate longer than broad, subsphaeroidal, with lateral margins broadly convex and posterior margin broadly convex, having a small triangular protuberance in the middle.

##### Coloration.

Body generally yellowish green, yellowish brown, or yellowish blue (Fig. 6). Antennae yellowish brown, with apical segments blackish brown. Eyes black to brown. Vertex with an edge-blurred dark or well-defined black longitudinal maculation (Fig. 6N, P). Postocular band broad and black, extending to posterior margin of lateral lobes of pronotum. Pronotum with a black longitudinal stripe in the middle that is equal to, or narrower but never broader than, the width of the yellow or yellowish-brown longitudinal stripe on both besides (Fig. 6N, P). Tegmen with three distinct, broad, longitudinal stripes, one in the middle and the other two at anterior and posterior margins. Hind wing dark or hyaline. Fore and middle legs green, yellowish green or yellowish brown; apex of the third tarsal segment, claws and arolium black in some individuals. Hind femur yellowish brown; knees black; upper surface with three distinct black spots, the basal one much smaller or even indistinct in some individuals; outer surface without any maculation. Hind tibia dark blue to blueish green; base, spurs and apical half or full length of tibial spines black. Tarsus pale yellowish blue or dark.

##### Measurements.

Male: BL: 25.0–29.5 mm; PNL: 6.5–7.3 mm; TL: 9.4–10.0 mm; HFL: 9.5–10.5 mm. Female: BL: 30.0–37.5 mm; PL: 9.0–9.5 mm; TL: 10.0–12.5 mm; HFL: 17.6–18.0 mm.

##### Distribution.

China (Hubei, Hunan, Guangxi, Guizhou, Chongqing, Sichuan, Yunnan), Vietnam.

##### Biology.

*Tonkinacris sinensis* occurs one generation each year and winters by egg. It lives in the bush and hassock of low hillside ~ 300–700 m a.s.l and is omnivorous, feeding on more than ten families of plants such as Compositae, Papilionaceae, Gramineae, Berberidaceae, and Meliaceae. It also heavily injures maize and sorghum and is one of the important pests of montane drought crops (Li and Xia 2006).

##### Remarks.

The most distinct differences between *Alulacris* and *Tonkinacris* are the position, length, color, and maculation of tegmina and the shape of the male cercus. While the tegmina of *A. nigristriatis* is separated dorsally in female (Fig. 3C–H), they are distinctly continuous dorsally in male (Fig. 3A). Furthermore, as shown in Figs 3, 6A, B, D, E, the paratype females of *A. nigristriatis* exhibit significant deformation, likely resulting from being preserved in small plastic tubes with ethanol when collected. This suggests that the apparent dorsal separation of the tegmina may be an artifact of improper preservation. Therefore, under normal conditions, the tegmina of *A. nigristriatis* may be dorsally contiguous in both sexes.

The most distinguishing characteristics separating *A. nigristriatis* from *A. shilinensis* are the presence of longitudinal stripes along the anterior, posterior, and central regions of the tegmina; the uniquely constricted apical shape of the cerci; and the mastoid furculae located centrally on the posterior margin of the tenth abdominal tergite. However, regardless of the position and length of tegmina, the mastoid furculae or the black stipes on tegmina, *A. nigristriatis* conforms more favorably to the generic diagnosis of *Tonkinacris* and shows no significant difference from *T. sinensis* (Fig. 6). Accordingly, we propose herein *A. nigristriatis* as a junior synonym of *T. sinensis*.

## ﻿Discussion

### ﻿Reliability of quantitative traits in defining new species

Speciation is the process that transforms demographically and genetically connected populations into divergent, reproductively isolated species (Waller et al. 2023). Many evolutionary processes can contribute to this transformation and most speciation events are likely caused by multiple divergence processes acting simultaneously (Butlin and Smadja 2018). Thus, it is necessary to identify the divergent traits among populations that are likely to contribute to reproductive isolation (phenotypic barriers) and characterize the evolutionary mechanisms and history of these traits (Ravinet et al. 2017).

Phenotypic traits can be grouped into two categories, quantitative traits and qualitative traits. A quantitative trait, also referred to as a quantitative character, is a measurable phenotype emerging from genetic and environmental factors that is distributed in magnitude in a population rather than all or none (Philibin and Crabbe 2015). Quantitative traits can be measured and expressed numerically, and contain wide ranges of phenotypes exhibited within a population, displaying much more phenotypic variation over a population than qualitative traits do. They are often referred to as “continuous” because they vary along a continuum. Quantitative traits can often be referred to as multifactorial because they are not only affected by multiple genes (polygenic), but also influenced by the external environment. Because quantitative traits are controlled by multiple genes, their genes do not typically have complete dominance but rather an additive and summative effect. Quantitative traits are imperative to studying the evolution of species populations. Because certain quantitative traits are more useful to survival, they are more likely to be passed on to future generation. Evolution and natural selection are better observed through quantitative rather than qualitative traits (https://study.com/learn/lesson/quantitative-traits-overview-examples.html).

Many quantitative traits have been hypothesized to influence the diversification dynamics of lineages over macroevolutionary time-scales (FitzJohn 2010; Harvey and Rabosky 2018). Sympatric speciation is possible even when fitness and mate choice depend on different quantitative traits (Kondrashov and Kondrashov 1999). Quantitative traits are not only the main focus when studying phenotypic variation distribution that exist within populations, but also often used for species identification in traditional morphological taxonomy. Once a quantitative trait is confirmed to contribute to speciation in a certain group, it can be used to distinguish different species (Christophoryová et al. 2016; Stiffler et al. 2018), and even define/delimit new species. There are lots of methods for continuous trait analysis (FitzJohn 2010, 2012; Beaulieu and O’Meara 2016; Harvey and Rabosky 2018) and species delimitation using data from quantitative traits (Tobias et al. 2010; Cadena et al. 2018; Douglas and Bouckaert 2022; Sukumaran and Meila 2024). However, for morphological taxonomists who are not familiar with molecular analysis and species delimitation approaches, they usually recognize the significance of difference in quantitative traits only based on their personal experience, but not as a rigorous statistical test. *Alulacris yanshanensis* was described based on only a single holotype female and three of the five differentiating characters are quantitative (Mao et al. 2011a). However, after a rigorous statistical test, it was found that there is no significant difference in interocular distance, the ratio of prozona and metazona and the ratio of width and length of tegmen between *A. yanshanensis* and *A. shilinensis* (Table 1). The previously reported difference in the condition of the lateral margins of frontal ridge is now confirmed to be a misinterpretation due to the lack of examining types of *A. shilinensis*. In fact, the lateral margins of frontal ridge are weakly expanded between antennae in both *A. yanshanensis* and *A. shilinensis* (Fig. 1A, B). As for the color of the ventral knee lobes of the hind femur, our observations indicate that it displayed some variation in *A. shilinensis* (Fig. 1E–N). Given the absence of significant difference between these two species, they should be considered as conspecific.

To reduce the likelihood of describing synonyms, it is recommended that new species should not be established based solely on very few specimens—nor on a single type. Furthermore, the diagnostic value of quantitative traits used to distinguish new species should be rigorously tested using statistical methods.

### ﻿Importance of the accuracy of generic assignment in determining new species

During the course of identification and classification, the first and most important procedure is to assign a specimen to a correct genus, and then the specimen will be compared in principle with all species within this genus to determine whether it is a known species or not. However, if it is assigned to an incorrect genus, then of course, the so-called distinct difference may be found from all known species of this genus and a junior synonym may be incorrectly determined and described as new. In grasshoppers, such examples have been observed in many species such as *Oxyoides wulingshanensis* Zheng & Fu, 1994, *Oxyoides bamianshanensis* Fu & Zheng, 1999, *Oxyoides longianchorus*Huang et al., 2007, *Caryandoides maguas* Zheng & Xie, 2007 (Huang et al. 2009), *Flatovertex cyaneitibialis* Zhang & Han, 2010, *Flatovertex rufotibialis* Zheng,1981, *Flatovertex nigritibialis* Zheng & Zhang, 2006 (Zheng et al. 2006; Huang et al. 2013a), *Longgenacris rufiantennus* Zheng & Wei, 2003 (Jiang et al. 2019), and *Longchuanacris guangxiensis* Zheng & Ren, 2007 (Mao et al. 2011b). Possibly more cases will be found in our future studies. Therefore, we have to be careful of such errors, and try our best to increase the accuracy of the generic assignment of a specimen and to minimize the description of synonyms as much as possible.

## Supplementary Material

XML Treatment for Alulacris shilinensis

XML Treatment for Tonkinacris sinensis

## ﻿Additional information

### Use of AI

No use of AI was reported.

### Author ORCIDs

Jianhua Huang https://orcid.org/0000-0001-9850-5119

### Data availability

All of the data that support the findings of this study are available in the main text.
